# Molecular Diagnosis of Pompe Disease in the Genomic Era: Correlation with Acid Alpha-Glucosidase Activity in Dried Blood Spots

**DOI:** 10.3390/jcm10173868

**Published:** 2021-08-28

**Authors:** Fanny Thuriot, Elaine Gravel, Katherine Hodson, Jorge Ganopolsky, Bojana Rakic, Paula J. Waters, Serge Gravel, Sébastien Lévesque

**Affiliations:** 1 Department of Pediatrics, Université de Sherbrooke, Sherbrooke, QC J1H 5H3, Canada; Fanny.Thuriot@USherbrooke.ca (F.T.); Elaine.Gravel@USherbrooke.ca (E.G.); Paula.J.Waters@USherbrooke.ca (P.J.W.); Serge.Gravel@USherbrooke.ca (S.G.); 2 Sherbrooke Genomic Medicine, Sherbrooke, QC J1H 5H3, Canada; 3 Dynacare, Laval, QC H7L 4S3, Canada; hodsonk@dynacare.ca (K.H.); ganopolskyj@dynacare.ca (J.G.); 4 BC Children’s Hospital, Vancouver, BC V6H 3N1, Canada; bojana.rakic@cw.bc.ca

**Keywords:** Pompe disease, gene panel sequencing, alpha-glucosidase, GAA, dried-blood spots

## Abstract

Measurement of alpha-glucosidase activity on dried blood spots has been the main method to screen for Pompe disease, but a paradigm shift has been observed in recent years with the incorporation of gene panels and exome sequencing in molecular diagnostic laboratories. An 89-gene panel has been available to Canadian physicians since 2017 and was analyzed in 2030 patients with a suspected muscle disease. Acid alpha-glucosidase activity was measured in parallel in dried blood spots from 1430 patients. Pompe disease was diagnosed in 14 patients, representing 0.69% of our cohort. In 7 other patients, low enzyme activities overlapping those of Pompe disease cases were attributable to the presence of pseudodeficiency alleles. Only two other patients had enzymatic activity in the Pompe disease range, and a single heterozygous pathogenic variant was identified. It is possible that a second variant could have been missed; we suggest that RNA analysis should be considered in such cases. With gene panel testing increasingly being performed as a first-tier analysis of patients with suspected muscle disorders, our study supports the relevance of performing reflex enzymatic activity assay in selected patients, such as those with a single *GAA* variant identified and those in whom the observed genotype is of uncertain clinical significance.

## 1. Introduction

Pompe disease is an autosomal recessive disorder caused by pathogenic variants in the *GAA* gene, which encodes an acid alpha-glucosidase enzyme [1]. This lysosomal glycogen storage disorder has a prevalence of 1:40,000 in individuals in the United States, with increased incidence in African Americans [2,3]. It may present at any age, from infancy to late adulthood [4]. Patients with infantile onset Pompe disease (IOPD) often present with hypotonia, respiratory insufficiency, and cardiomyopathy, resulting in death in the first year of life if untreated. Patients with late onset Pompe disease (LOPD) present with a slowly progressive phenotype, including muscle weakness, mainly limb-girdle, and respiratory failure [5]. Most adult patients share the common c.-32-13T>G leaky splicing variant [6]. Because of the variable severity of this disease, its rarity, and the extensive differential diagnoses, diagnosis is typically made years after the onset of symptoms [7,8]. In 2006, enzyme replacement therapy was first approved to treat patients with Pompe disease [9]. To be effective, the treatment must be administered as soon as possible, as delay may cause irreversible damage [10]. In that context, newborn screening was proposed to enable early diagnosis. Several pilot studies subsequently demonstrated its favorable impact on patient outcomes, leading to the introduction of population newborn screening for Pompe disease in some jurisdictions [11,12]. To provide a low-cost and rapid screening test, dried blood spots (DBS) are used to measure acid alpha-glucosidase enzymatic activity, by fluorometric assays or by tandem mass spectrometry [11,13]. However, pseudodeficiency alleles can generate false-positive results, as these benign hypomorphic variants cause a decrease in the observed enzymatic activity, but without causing Pompe disease. To confirm diagnosis, sequencing analysis of the *GAA* gene must be performed [14,15].

With decreasing cost of sequencing technologies, targeted and whole-exome sequencing have increasingly been used to diagnose patients with Pompe disease, especially in adults with suggestive symptoms or in populations where newborn screening is not yet available. These methods have been shown to have a high diagnostic yield in contexts where differential diagnoses, such as other limb-girdle muscular dystrophies (LGMD), cannot be discounted [16,17,18].

Here we present data supporting the use of acid alpha-glucosidase enzymatic assay on dried blood spot as a reflex test following molecular analysis, for the confirmation of diagnosis of Pompe disease in symptomatic patients.

## 2. Materials and Methods

Clinical molecular testing was performed on a total of 2030 pediatric and adult patients with a suspected muscle disorder who were followed in outpatient clinics across Canada (general neurology, specialized neuromuscular, clinical genetics). To be eligible for testing, the patient had to have no reported diagnosis explaining their phenotype and to present weakness (any pattern) or symptom(s) suggestive of muscle involvement (i.e., myalgia, rhabdomyolysis, exercise intolerance, or unexplained respiratory insufficiency). Most patients (93.5%) also presented at least one abnormal laboratory finding suggestive of muscle involvement (plasma creatine kinase (CK), EMG, muscle biopsy, or MRI). Demographics and clinical information were obtained from the laboratory requisition [18]. Genetic counselling and follow up tests were recommended, when appropriate, to the referring physician. Patients were referred to different specialized genetic services across the country, and additional genetic counselling support was available at Dynacare (Laval, QC, Canada) by phone or virtual consultation when needed.

### 2.1. Acid Alpha-Glucosidase Enzymatic Activity Assays

Dried blood spots (DBS) were collected for measurement of acid alpha-glucosidase activity at either Dynacare laboratory (Laval, QC, Canada) or the BC Children’s Hospital (Vancouver, BC, Canada). Both laboratories used similar fluorometric assays, based on a previously published methodology [13] with minor modifications. These assays rely upon enzymatic cleavage of the alpha-glucosidase substrate 4-methylumbelliferyl-alpha-D-glucopyranoside (4-MUG) at acidic pH in the presence of acarbose, which inhibits potentially interfering isoenzymes, such as maltose-glycoamylase. After stopping the enzymatic reaction by addition of a strongly alkaline buffer, fluorescence of the free 4-methylumbelliferone (4-MU) reaction product is measured, and its concentration calculated using a 4-MU calibration curve. Both laboratories also assayed at least one other enzyme in parallel (data not shown), which provided a control for specimen quality. Further information on assay procedures specific to each laboratory are summarised as follows.

At Dynacare, an extract containing the enzyme was eluted from a 3 mm DBS punch with 40 mM sodium acetate buffer (pH 3.8) for 1 h at 4 °C, then incubated with 4-MUG for 20 h at 37 °C in the presence of acarbose. Fluorescence was read with a fluorometer with excitation at 355 nm and emission at 460 nm, within one hour after stopping the enzymatic reaction with 150 mM EDTA, pH 11.3–12. Acid alpha-glucosidase enzymatic activity was expressed in pmol/hour/punch. Cut-off values were 4.49 and 5.39 pmol/hour/punch for “reduced” and “borderline” enzymatic activities, respectively. Values above 5.39 pmol/hour/punch were considered normal.

At BC Children’s Hospital, two 3 mm DBS punches from each specimen were extracted with 400 µL of deionized water in a 1.5 mL microcentrifuge tube. Each sample was vortexed for 10 s followed by gentle mixing at room temperature for 1 h on a rocking platform. After 1 h the tubes were spun in a refrigerated centrifuge (11,600 rpm, 5 min). The working substrate solution was 2.8 mM 4-MUG in 40 mM sodium acetate buffer, pH 3.8, with 15 µM acarbose. Enzyme reactions consisted of 32 µL DBS extract and 48 µL substrate solution. The reactions were incubated overnight for 20 h at 37 °C in a PCR machine. DBS extracts for blanks were also incubated. After 20 h the reaction was stopped by adding 160 µL of 10 mM NaOH stop buffer, pH 10.5, to all tubes including blanks. Fluorescence was measured in a 96-well plate (Synergy 2 microplate reader). Results for acid alpha-glucosidase activity were reported with a normal reference range of 2.0–9.4 pmol/h/µL).

Since enzymatic activity assays for patients in this study were performed by two different laboratories, we normalized each reported value relative to the corresponding lower limit of the normal reference range. We do not have DBS values for all patients who underwent gene panel testing because parallel enzymatic testing was not supported at the beginning of the program. Enzyme activity results were available for 1430 patients (1314 measured at Dynacare; 108 measured at BC Children’s Hospital), representing 70.4% of the study cohort.

### 2.2. Gene Panel and Next Generation Sequencing Method

Blood samples were collected to extract genomic DNA using a MagnaPure instrument (Roche, MA). A clinical gene panel test was performed at the Sherbrooke Genomic Medicine laboratory (a not-for-profit organization), and the cost of the test was covered by a special program with financial support from Sanofi Genzyme Canada. This gene panel included the *GAA* gene for Pompe disease, and genes with muscle-associated disorders, mainly limb-girdle muscular dystrophies, but also congenital muscular dystrophies, congenital myasthenic syndromes, nemaline myopathy, myofibrillar myopathy, centronuclear myopathy, collagen VI–related myopathies, inclusion myopathies, metabolic myopathies, rigid spine syndromes, and scapuloperoneal syndromes [18]. DNA libraries were prepared according to standard protocol (Kapa Biosystems, Roche, MA), following targeted capture (xGen Predesigned Gene Capture Pools, Integrated DNA Technologies, Kanata, ON, Canada, and sequencing on a NextSeq 550 (Illumina, CA, USA) sequencer with a 150-bp paired-end protocol.

### 2.3. Splicing Analysis

Blood sample was collected in Tempus Blood RNA Tubes and RNA was extracted using MagMAX™ for Stabilized Blood Tubes RNA Isolation Kit, according to standard protocol (ThermoFisher, Waltham, MA, USA). RNA integrity was assessed with an Agilent 2100 Bioanalyzer (Agilent Technologies, Saint-Laurent, QC, Canada). Reverse transcription was performed on 750 ng total RNA with Transcriptor reverse transcriptase, random hexamers, dNTPs (Roche Diagnostics, Laval, QC, Canada), and 10 units of RNAse OUT (Invitrogen, Waltham, MA, USA) following the manufacturer’s protocol in a total volume of 10 µL. All forward and reverse primers were individually resuspended to 20–100 μM stock solution in Tris-EDTA buffer (IDT) and diluted as a primer pair to 1.2 μM in RNase DNase-free water (IDT). End-point PCR reactions were done on 10 ng cDNA in 10 μL final volume containing 0.2 mmol/L each dNTP, 0.6 μmol/L each primer, and 0.2 units of TransStart FastPfu Fly DNA Polymerase (Trans). An initial incubation of 2 min at 95 °C was followed by 35 cycles at 95 °C 20 s, 55 °C 20 s, and 72 °C 60 s. The amplification was completed by a 5-min incubation at 72 °C. PCR reactions are carried on thermocyclers C1000 Touch Thermal cycler (Bio-Rad), and the amplified products were analyzed by automated chip-based microcapillary electrophoresis on Labchip GX Touch HT instruments (Perkin Elmer, Woodbridge, ON, Canada). Amplicon sizing and relative quantitation was performed by the manufacturer’s software, before being uploaded to the LIMS database.

### 2.4. Bioinformatics

We analyzed the sequencing data using a Linux-based bioinformatics pipeline based on the one developed by the McGill University and Genome Quebec Innovation Centre (bitbucket.org/mugqic/mugqic_pipelines (accessed on 14 June 2021)) as previously described [18]. Filtered variant lists obtained from the bioinformatics pipeline were then interpreted with an inhouse script and manual revision. Deletion and duplication analysis were performed using the CoNVaDING software [19] and manual review of binary alignment map files before quantitative PCR confirmation using Taqman Copy Number Assay (ThermoFisher Scientific, Montreal, QC, Canada). The recurrent *GAA* exon 18 deletion could be detected with both visual inspection of the sequencing reads and the CoNVaDING software, using a previously known positive control [17]. Variants were revised manually and were reported according to the American College of Medical Genetics and Genomics guidelines [20].

### 2.5. Statistical Analysis

Statistical analysis was performed using GraphPad Prism version 8.2.0 for Windows, GraphPad Software, San Diego, CA, USA, www.graphpad.com (accessed on 21 June 2021).

## 3. Results

### 3.1. Patients with Pompe Disease

A molecular diagnosis was identified in 272 of the 2030 patients. Of these, 14 patients were diagnosed with Pompe disease, representing 5.1% of all diagnoses, 1.15% of our LGMD patients, and 0.69% of our entire cohort. All 14 Pompe disease patients were found to be compound heterozygous for two variants, each considered pathogenic or likely pathogenic [20], in the *GAA* gene.

Among these patients with Pompe disease, 3 had IOPD, ranging from 1 month-old to 2 years-old. The 11 remaining patients had LOPD, ranging from 33 to 68 years-old (mean, 50 years-old). All patients with LOPD carried the c.-32-13T>G variant in a compound heterozygous state, whereas only one infantile onset patient carried the c.-32-13T>G variant. Most variants were identified in only one patient. All reported causal variants found in these patients are listed in Table 1. The c.1805C>T (p.Thr602Ile) variant was not previously known to be disease-causing, but was reclassified as likely pathogenic based on the very low acid alpha-glucosidase activity and the presence of a second pathogenic variant in a patient with phenotype consistent with IOPD. Later, the two variants were shown to be *in trans* following parental study.

Patients with IOPD presented with hypotonia or limb-girdle weakness. One among the three IOPD cases did not show cardiomyopathy at the time of diagnosis.

All but one LOPD patient presented with limb-girdle weakness, and 5 of them presented with respiratory insufficiency as well. A single LOPD patient presented only with unexplained respiratory insufficiency and showed normal CK levels and EMG.

Among all patients with available CK data (12), only one patient (LOPD) had normal CK at the time of diagnosis.

### 3.2. Enzymatic Activity and Genotype

**Figure 1:**
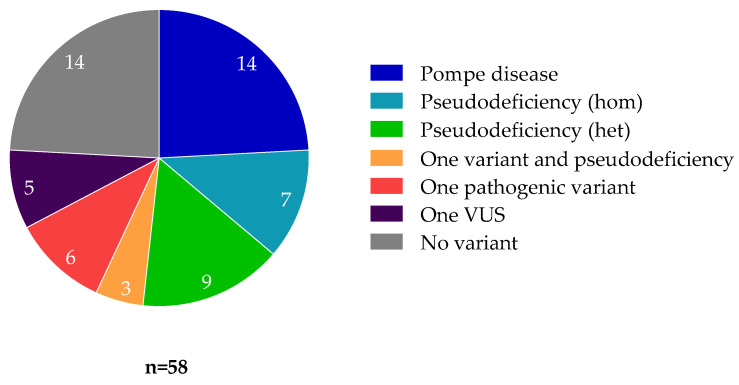
Genotype-based categories of patients with decreased acid alpha-glucosidase activity. Pompe disease—2 pathogenic or likely pathogenic *GAA* variants identified. Pseudodeficiency (hom)—One known pseudodeficiency allele was identified in a homozygous state, in the absence of any other identified variant. Pseudodeficiency (het)—One known pseudodeficiency allele was identified in a heterozygous state, in the absence of any other identified variant. One variant + pseudodeficiency—A single variant (pathogenic or of uncertain significance was identified in a heterozygous state, together with at least one known pseudodeficiency allele. One pathogenic variant—A single pathogenic or likely pathogenic variant was identified. One VUS—A single heterozygous variant of uncertain significance was identified. No variant—No variant of any kind was identified in the *GAA* gene.

Among our cohort of 2030 patients, results of acid alpha-glucosidase activity were available for 1430 patients. Overall, 58 of the 1430 patients (4.1%) had a decreased enzymatic activity (Figure 1). Of those, only 14 (24.1%) had two pathogenic or likely pathogenic variants confirming the diagnosis of Pompe disease. Fourteen (24.1%) patients did not harbor any variant which could explain this decreased enzymatic activity. Conversely, all Pompe disease patients identified through the gene panel had a low enzymatic activity, as expected.

**Figure 2:**
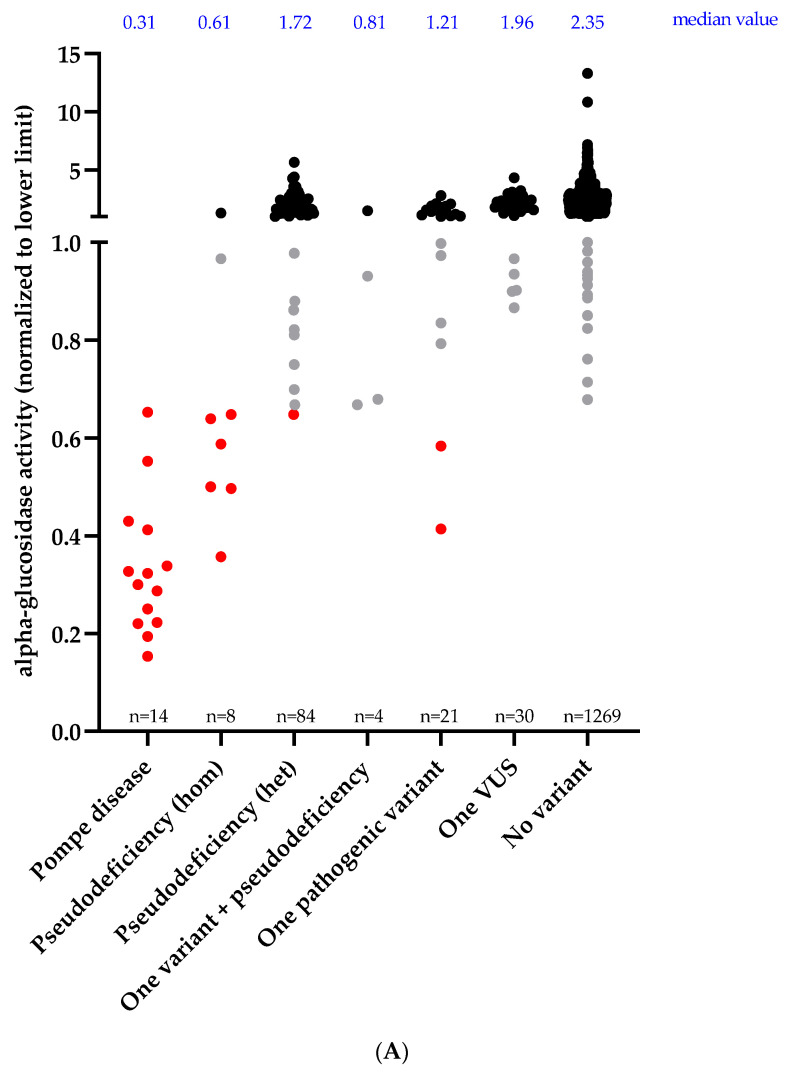

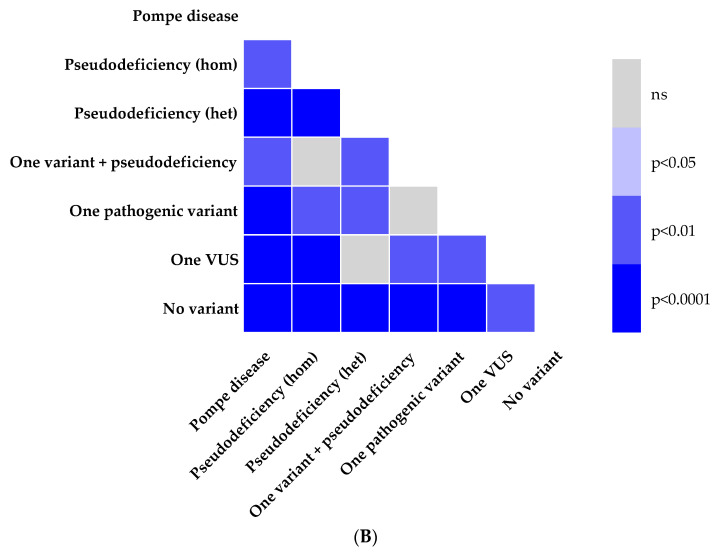
Acid alpha-glucosidase activity and genotypes of our cohort (*n* = 1430). Since enzymatic activity was assayed in two different centres, activity is reported normalized to the lower limit of the normal reference range according to the corresponding laboratory. (**A**) Black: normal enzymatic activity (>1.00); grey: borderline enzymatic activity (0.65–1.00); red: decreased enzymatic activity corresponding to the levels observed in Pompe disease (<0.65). The median value of each subgroup is reported in blue. (**B**) *p* values calculated using Mann–Whitney test. ns = not significant.

Enzymatic activities, grouped according to the patients’ *GAA* genotype categories, and the corresponding statistical comparisons using a Mann–Whitney test, are illustrated in Figure 2. The decreasing order of median values for enzymatic activities was as follows: no variant (2.35) > one VUS (1.96) > pseudodeficiency het (1.72) > one pathogenic variant (1.21) > one variant + pseudodeficiency (0.81) > pseudodeficiency hom (0.61) > Pompe disease (0.31). Enzymatic activity of patients with Pompe disease were statistically different from all other classes of genotype, including pseudodeficiency. Pompe disease patients had an enzymatic activity of less than 0.65 normalized activity, meaning less than 65% of activity of the lower reference limit.

Notably, 9 other patients had an enzymatic activity lower than 0.65, including 7 patients with pseudodeficiency alleles and 2 patients with a single pathogenic variant (c.-32-13T>G) identified. Of the 7 patients with pseudodeficiency alleles, 6 harboured these variants in the homozygous state and one in the heterozygous state. Enzymatic activity measurement was repeated for one of the patients with a single pathogenic variant, on a new specimen, giving a similar result. To exclude a possible second variant not detected by DNA sequencing of coding sequences, RNA studies were suggested to check for potential splicing defects (reflecting a deep intronic splicing variant) for the patient with repeated low enzymatic activity. However, the suggestion was declined by the referring physician, who considered that the patient’s evolution was not suggestive of Pompe disease. Splicing studies could be performed on the second patient with a single pathogenic variant but no splicing defect was detected. Ultimately, none of these two patients received a diagnosis of Pompe disease nor did they have access to enzyme replacement therapy. Detailed clinical description of these patients was not available to us, therefore we could not exclude the possibility of Pompe disease. For comparison, we note that 8 other patients found to be heterozygous carriers of the c.-32-13T>G variant had normal enzymatic activity (>1.00), while 2 others showed “borderline” enzyme results (normalized activities between 0.65 and 1.00).

## 4. Discussion

Measurement of acid alpha-glucosidase activity on dried blood spots is used for newborn screening and has also been used widely in “high-risk” populations to screen for Pompe disease [11,12,21]. Although it is an efficient and low-cost approach to identify potential cases of Pompe disease, putative positive cases require confirmation by mutation analysis and exclusion of pseudodeficiency alleles [22]. The increased availability of gene panel and whole-exome sequencing is changing clinical practice, as these methods can address the wide differential diagnoses that are facing clinicians during their investigations of patients with muscle weakness. Measurement of acid alpha-glucosidase activity as a first-tier test in this context is therefore being questioned. In this study, we show the relevance of combining sequencing and enzymatic assays to avoid missing a diagnosis of Pompe disease or to clarify the implications of an observed variant of uncertain significance.

In our cohort, Pompe disease showed a prevalence of 0.69%, and accounted for 1.15% of patients with limb-girdle weakness. The clinical presentation of the 14 patients identified with Pompe disease is similar to historical cohorts and, not surprisingly, all LOPD cases carried the common c.-32-13T>G variant [23,24]. Table 2 provides a review of recent sequencing studies (2015–2021) which included more than 50 patients with muscle disorders. These studies cover wider genetic approaches such as whole-exome sequencing (WES) as well as targeted sequencing of gene panels [25,26,27,28,29,30,31,32,33]. The proportion of Pompe disease patients identified in our study is similar to previous studies, although it is slightly lower than the mean value of 1.27%. Variability in prevalence between studies is likely to be explained by differences in recruitment criteria. Indeed, some cohorts included only patients with limb-girdle muscular weakness, which is known to be the main type of weakness in patients with Pompe disease [34]. Since our cohort included other types of weakness, such as predominant distal weakness, it tended to incorporate patients with a broader spectrum of muscular disorders and thus dilute the proportion of Pompe patients. The higher proportion of 1.15% of Pompe disease within the group of patients with limb-girdle weakness supports this hypothesis. Finally, it is interesting to note that the rate of Pompe disease identified by these sequencing studies and our in patients presenting with limb-girdle weakness is similar to previous studies screening with measurement of acid alpha-glucosidase activity [15,35,36]. This suggests that if cases are missed by first-tier sequencing approaches (thus presumed to be heterozygous carriers), it is likely to represent a small proportion a priori.

Our study provides a direct comparison between results of sequencing and enzymatic activity measurement. Acid alpha-glucosidase activity was assayed using dried blood spots and results were available for 1430 patients, representing 70.4% of our entire cohort. A total of 58 patients had decreased acid alpha-glucosidase activity, with Pompe disease accounting for a quarter of the patients with decreased enzymatic activity (<1.00 normalized activity). Another quarter was explained by pseudodeficiency. In a third quarter, only a single heterozygous *GAA* variant (pathogenic or of uncertain significance, together with a co-existing pseudodeficiency allele in some cases) was found. Finally, the last quarter was composed of patients without any detected *GAA* variant. However, by setting the threshold at 0.65 normalized activity, we showed that only 23 patients had enzymatic values falling within the range corresponding to Pompe disease. Fourteen were explained by molecularly confirmed Pompe disease, while seven were explained by the presence of one or more pseudodeficiency alleles, which are known to reduce enzymatic activity without causing clinical disease [15]. This and data from Figure 2 illustrate well the difficulty in differentiating homozygous pseudodeficiency alleles from Pompe disease cases based on enzyme activity alone. The two remaining low values represented two patients with only one pathogenic variant (c.-32-13T>G) identified; one whose enzymatic activity was confirmed to be low on a second specimen. In both cases, we were not able to confirm a diagnosis of Pompe disease. Either we did not have the opportunity to perform RNA studies to exclude the possibility that a second causal variant could have been missed, or the results of these studies did not suggest the presence of second variant. An example of such a deep intronic variant has been reported in a Pompe disease patient previously [37]. Although whole-genome sequencing has the potential to identify deep intronic variants, RNA studies and enzymatic activity measurement are likely to be required as reflex testing to confirm bioinformatic predictions of splicing events. The scarce availability of whole-genome sequencing and RNA sequencing in clinical laboratories probably explain why deep intronic variants are currently absent from the Pompe disease variants database [38]. Other factors such as promoter hypermethylation could be considered, and muscle biopsy remains helpful in absence of a second variant to support a diagnosis of Pompe disease by showing the pathogenic accumulation of glycogen.

Enzymatic activity measurement as a reflex test can also be used to reclassify variants of uncertain significance in certain circumstances. In particular, as illustrated by our patient carrying a pathogenic variant and the variant of uncertain significance c.1805C>T (p.Thr602Ile), a low enzyme activity consistent with Pompe disease may contribute to reclassify the latter as likely pathogenic, according to ACMG guidelines [20]. Indeed, ClinGen’s expert panel has been working on a modified version of the ACMG guidelines for improvement of classification of variants in the *GAA* gene, and includes acid alpha-glucosidase activity in their interpretation in a variety of scenarios [39]; [https://clinicalgenome.org/site/assets/files/3969/clingen_lsd_acmg_specifications_v1.pdf (accessed on 22 June 2021)].

We conclude that a combined approach, using DNA sequencing followed by dried blood spot acid alpha-glucosidase activity assay as a reflex test when indicated, can be recommended as best practice to identify Pompe disease patients in the molecular era. Although the rate of diagnosis of Pompe disease is similar in LGMD patients using gene panels or WES compared to enzymatic screening, reflex enzymatic testing potentially decreases the risk of missing a diagnosis when only one pathogenic variant is detected by DNA sequencing, and may also be used to confirm or rule out a diagnosis of Pompe disease following the observation of a genotype of uncertain clinical significance.

## Figures and Tables

**Table 1:** Pathogenic and likely pathogenic variants found in the *GAA* gene in patients with Pompe disease. All variants were observed only in the compound heterozygous state. Occurrence denotes the number of times each variant was observed in this group (14 patients; 28 alleles).

Pathogenic/Likely Pathogenic Variants	Occurrence
c.32-13T>G	12
c.655G>A (p.Gly219Arg)	2
c.1115A>T (p.His372Leu)	2
c.1-?_2859+?del (p.Met1_Cys952del)	1
c.258dupC (p.Asn87fs)	1
c.525delT (p.Glu176fs)	1
c.706delG (p.Val236fs)	1
c.896T>C (p.Leu299Pro)	1
c.1396dupG (p.Val466fs)	1
c.1551+1G>C	1
c.1805C>T (p.Thr602Ile)	1
c.1912G>T (p.Gly638Trp)	1
c.1927G>A (p.Gly643Arg)	1
c.2242dupG (p.Glu748fs)	1
c.2577G>A (p.Trp859*)	1

**Table 2:** Literature review of cohorts including patients with Pompe disease.

Reference	Genetic Approach	Cohort	Number of Patients	Proportion of Pompe Patients	Recruitment
Ghaoui et al., 2015 PMID: 26436962 [30]	WES	LGMD	100	1.00%	Australia, retrospective research, muscle biopsies
Reddy et al., 2017 PMID: 27708273 [26]	WES	LGMD	55	1.82%	United States, research protocol
Chakravorty et al., 2020 PMID: 33250842 [27]	WES	Myopathy	201	1.00%	India, tertiary care hospital
**Mean proportion of Pompe disease patients (whole-exome sequencing)**				**1.27%**	
Töpf et al., 2020 PMID: 32528171 [31]	WES (429 genes analyzed)	LGMD and/or elevated CK	1001	1.00%	Europe and Middle East, 43 neuromuscular referral centers
Johnson et al., 2017 PMID: 29149851 [25]	WES (169 genes analyzed)	LGMD and/or elevated CK	606	1.98%	England, referred by clinicians
Nallamilli et al., 2018 PMID: 30564623 [28]	Targeted sequencing (35 genes)	LGMD	4656	0.82%	United States, Emory Genetics Laboratory
Savarese et al., 2018 PMID: 29880332 [32]	Targeted sequencing (93 genes)	LGMD, myopathies and/or isolated hyperCKemia	504	3.17%	Italy, tertiary centers for neuromuscular disorders
Bevilacqua et al., 2020 PMID: 31931849 [29]	Targeted sequencing (10 genes)	LGMD	2103	0.43%	Latin America, 20 institutions
Winder et al., 2020 PMID: 32337338 [33]	Targeted sequencing (266 genes)	Cardiomyopathy/skeletal muscle, muscular dystrophy, neuromuscular disorders, LGMD	6493	0.25%	United States, Invitae Laboratory
**Mean proportion of Pompe disease patients (targeted sequencing)**				**1.28%**	
This study	Targeted sequencing (89 genes)	Suspected muscle disorders	2030	0.69% (entire cohort) 1.15% (LGMD)	Canada, outpatient clinics

## Data Availability

Anonymized data will be shared by request from any qualified investigator. When not possible, given the risk to identify rare patients, additional aggregate data in table form will be produced to address specific questions.
